# Radioembolization and the Dynamic Role of ^90^Y PET/CT

**DOI:** 10.3389/fonc.2014.00038

**Published:** 2014-02-27

**Authors:** Alexander S. Pasciak, Austin C. Bourgeois, J. Mark McKinney, Ted T. Chang, Dustin R. Osborne, Shelley N. Acuff, Yong C. Bradley

**Affiliations:** ^1^The University of Tennessee Medical Center, Knoxville, TN, USA; ^2^The University of Tennessee Graduate School of Medicine, Knoxville, TN, USA; ^3^Mayo Clinic, Jacksonville, FL, USA; ^4^University of Virginia Medical Center, Charlotte, VA, USA

**Keywords:** radioembolization, ^90^ Y PET, radioembolization dosimetry, quantitative imaging, post-treatment imaging

## Abstract

Before the advent of tomographic imaging, it was postulated that decay of ^90^ Y to the 0^+^ excited state of ^90^Zr may result in emission of a positron–electron pair. While the branching ratio for pair-production is small (~32 × 10^−6^), PET has been successfully used to image ^90^ Y in numerous recent patients and phantom studies. ^90^ Y PET imaging has been performed on a variety of PET/CT systems, with and without time-of-flight (TOF) and/or resolution recovery capabilities as well as on both bismuth-germanate and lutetium yttrium orthosilicate (LYSO)-based scanners. On all systems, resolution and contrast superior to bremsstrahlung SPECT has been reported. The intrinsic radioactivity present in LYSO-based PET scanners is a potential limitation associated with accurate quantification of ^90^ Y. However, intrinsic radioactivity has been shown to have a negligible effect at the high activity concentrations common in ^90^ Y radioembolization. Accurate quantification is possible on a variety of PET scanner models, with or without TOF, although TOF improves accuracy at lower activity concentrations. Quantitative ^90^ Y PET images can be transformed into 3-dimensional (3D) maps of absorbed dose based on the premise that the ^90^ Y activity distribution does not change after infusion. This transformation has been accomplished in several ways, although the most common is with the use of 3D dose-point-kernel convolution. From a clinical standpoint, ^90^ Y PET provides a superior post-infusion evaluation of treatment technical success owing to its improved resolution. Absorbed dose maps generated from quantitative PET data can be used to predict treatment efficacy and manage patient follow-up. For patients who receive multiple treatments, this information can also be used to provide patient-specific treatment-planning for successive therapies, potentially improving response. The broad utilization of ^90^ Y PET has the potential to provide a wealth of dose–response information, which may lead to development of improved radioembolization treatment-planning models in the future.

## Introduction

Yttrium-90 (^90^ Y) has many properties that make it an ideal radionuclide for targeted internal therapy. It is one of a handful of radionuclides that is considered to be a pure β-emitter, emitting no gamma rays at any appreciable yield following decay. ^90^ Y also releases a higher energy electron than other pure β-emitters that have been used in internal therapy, with a maximum and average energy of 2.28 and 0.934 MeV, respectively. While ^90^ Y has found broad utility in targeted internal therapies ranging from radioimmunotherapy for non-Hodgkin’s lymphoma to radioembolization for hepatic malignancy, it is radioembolization that benefits most from the relatively high-energy of the ^90^ Y β-emission. The range of the maximum energy ^90^ Y β-particle is 11 mm in tissue, while its average energy β has a range slightly <4 mm. The penetration depth of the high-energy ^90^ Y β-particle is a key component of this radionuclide’s success in radioembolization, allowing for high dose deposition into the tissues between embolized capillaries.

As a pure β-emitter, ^90^ Y radioembolization has the advantage of a negligible radiation burden to both non-embolized portions of the liver and extra-hepatic tissues. Further, patients can be released shortly after treatment with minimal precautions due to both the lack of appreciable gamma emissions and the lack of free ^90^ Y, which may be transferred to others by exposure to body fluid. Unfortunately, these benefits come at a cost, as conventional direct imaging of this radionuclide is not possible.

The standard-of-care post-treatment imaging of ^90^ Y radioembolization is indirect imaging using bremsstrahlung SPECT with a wide energy window. Despite its common use, this method presents a number of drawbacks. Namely, the image quality produced by ^90^ Y bremsstrahlung SPECT is lower than that produced from conventional SPECT imaging radiotracers. Quantification of bremsstrahlung SPECT images also requires specialized techniques (1–3), making it impractical in the typical clinical setting.

Recently, PET has been identified as a new method of directly visualizing the activity distribution of ^90^ Y radioembolization. Imaging is based on a minor decay branch of ^90^ Y resulting in the generation of annihilation photons.

## Physics Behind ^90^Y and Emission of Annihilation Photons

In 1955, Johnson et al. (4) and Ford (5) independently found evidence of a 0^+^ first excited state in ^90^Zr, albeit in different ways. Ford (5) predicted this state theoretically, while Johnson et al. (4) measured the positron emission resulting from the de-excitation of this state experimentally using a magnetic spectrometer. Decay of ^90^ Y to the first excited state of ^90^Zr 0^+^ occurs rarely, but will result in subsequent de-excitation of ^90^Zr with several different possible emissions. A monopole transition of ^90^Zr 0^+^ → 0^+^ occurs by either the emission of a monoenergetic 1.75-MeV conversion electron or, because the energy of the ^90^Zr 0^+^ excited state exceeds 1.022 MeV, by internal pair-production (6, 7). Unlike typical excited states, in a 0^+^ → 0^+^ monopole transition the emission of a gamma ray is absolutely forbidden (6).

Following publication of Johnson’s findings (4), Greenberg and Deutsch (8) used a magnetically focused coincidence detector to accurately describe the ratio for positron emission of several β-radionuclides that were traditionally considered to be pure β-emitters. Greenberg determined the branching ratio for positron emission of ^90^ Y to be 36 ± 0.9 × 10^−6^.

More recently, Selwyn et al. (9) was able to experimentally determine the ^90^ Y branching ratio for positron emission with more accuracy than previous reports. A high purity coaxial germanium detector was used along with Monte Carlo modeling to identify the annihilation peak, and subtract out both the bremsstrahlung and environmental background (9). Selwyn identified the probability of positron emission per decay of ^90^ Y to be 31.86 ± 0.47 × 10^−6^ (9).

A more detailed explanation of the physics of positron emission resulting from the ^90^Zr 0^+^ → 0^+^ transition is given by D’Arienzo (10).

## First Images

While the presence of a ^90^Zr 0^+^ excited state and resultant minor branch for positron emission following decay of ^90^ Y has been known for over half of a century, coincidence imaging has been performed only in the past few years. In 2004, Nickles et al. (11) performed a quantitative measurement of annihilation photons emitted from a ^90^ Y source using a sodium-iodide scintillation detector pair operating in fast coincidence. The small branching ratio of ^90^ Y was measured with high precision with a trues:randoms ratio of 1000:1. Nickles also obtained some of the first phantom images with a micro-Derenzo phantom filled with a 50-MBq/mL ^90^ Y activity concentration on a Concorde microPET scanner. The scanner’s excellent resolution easily resolved phantom objects as small as 1.6 mm in size. While the true-coincidence count-rate measured by Nickles was low, he proposed that ^90^ Y coincidence imaging may prove clinically useful due to the possibility of accurate post-infusion quantification (11).

In 2009, Lhommel et al. (12) performed one of the first post-infusion clinical ^90^ Y PET/CT scans. Images were acquired using a Phillips Gemini Time-of-flight (TOF) system. A 2.5-mm thick copper ring was fabricated and placed over the detectors to decrease the count-rate from lower energy bremsstrahlung and prevent detector saturation. Qualitative comparison with bremsstrahlung SPECT showed superior resolution and a microsphere distribution that closely matched areas of high metabolic uptake seen on ^18^ FDG PET/CT imaging.

Following these two preliminary reports describing the possible viability of ^90^ Y PET/CT imaging, multiple authors have presented results from both patient and phantom imaging obtained on many different scanner types. Table 1 summarizes the PET/CT systems used in selected phantom studies from the literature.

**Table 1:** **Published ^90^ Y PET phantom imaging summary**.

Reference	Scanner	Detector material	Reconstruction parameters	Count-rate linearity^a^ (GBq)	Resolution (mm)
Lhommel et al. (13)	Philips Gemini TF	LYSO	2i33s, TOF, RR^b^	2.2	–
Werner et al. (14)	Siemens BioGraph Hi-Rez 16	LSO	8i16s	–	6.4
van Elmbt et al. (15)	Philips Gemini TF	LYSO	3i8s, TOF	2.3	9.3
van Elmbt et al. (15)	Philips Gemini Power 16	GSO	3i8s, 8 mm Gaussian filter	2.3	10.0
van Elmbt et al. (15)	Siemens Ecat Exact HR^b^	BGO	3i8s, 8 mm Gaussian filter	1.2	10.6
Willowson et al. (16)	Siemens BioGraph mCT	LSO	1i21s, TOF, RR	4.6	–
Bagni et al. (17)	GE Discovery ST	BGO	2i15s, RR	2.0	6.5
D’Arienzo et al. (18)	GE Discovery ST	BGO	3i	–	5.0
Elschot et al. (19)	Siemens BioGraph mCT	LSO	3i21s, TOF, RR	–	–
Kao et al. (20)	GE Discovery 690	LYSO	3i18s, TOF, RR	3.2	10.0

*^a^Count-rate linearity found up to the total ^90^ Y activity listed*.

*^b^Applied post-reconstruction using a non-vendor supplied method*.

## Confounding Factors

^90^ Y is a poor PET radiotracer when compared to those routinely used for diagnostic purposes. Aside from the low true-coincidence count-rate due to the small branching ratio for positron emission, several other factors may contribute to the difficulty of ^90^ Y PET imaging. These factors can be broken into two broad categories: those associated with the low true-coincidence rate and those associated with the high singles rate due to bremsstrahlung X-rays. These issues are introduced here and discussed through the remainder of this article.

### The low true-coincidence rate

The low true-coincidence rate in ^90^ Y PET imaging results in long scan times and noisy images, which are expected challenges given the low branching ratio for ^90^ Y positron emission (11). Additionally, there are also several less obvious challenges associated with the low true-coincidence rate. Modern PET/CT scanners use detectors composed primarily of either cerium-doped lutetium yttrium orthosilicate (LYSO) or cerium-doped lutetium orthosilicate (LSO). LYSO has become the scintillator of choice for state-of-the-art PET systems, especially as TOF becomes standardized due to both a superior energy resolution to bismuth-germanate (BGO) and a light emission decay rate, which is nearly an order of magnitude faster. Both LSO and LYSO contain lutetium which, based on natural isotopic abundance contains 2.59% ^176^Lu (13). ^176^Lu beta decays with a long half-life, emitting several prompt gamma rays, one of which has an energy of 401 keV. This 401-keV gamma ray falls within the typical PET energy window resulting in the introduction of a low background randoms rate. The typical true-coincidence count-rates associated with conventional PET radiotracers are orders of magnitude higher than that created by the presence of natural ^176^Lu, thus allowing this intrinsic radioactivity to be effectively ignored. Unfortunately, this may not be the case for ^90^ Y PET imaging and this intrinsic activity could have implications affecting both the accuracy of quantification and image quality.

### Bremsstrahlung X-rays

The infused ^90^ Y dosage in radioembolization can range anywhere from 1 to 4 GBq, and all of this activity can easily be within the axial field of view (FOV) of a single PET bed position. Since every decay of ^90^ Y emits a high-energy β, a large fluence of bremsstrahlung X-rays can be produced. In fact, in ^90^ Y PET imaging the singles count-rate from bremsstrahlung exceeds the true-coincidence count-rate by a large margin. The ^90^ Y bremsstrahlung energy spectrum is broad (21) and produces the highest photon yield at energies below 20 keV. Fortunately, the majority of these low-energy photons are likely to be significantly attenuated by the patient. The spectrum of released photons is relatively constant from 20 to 500 keV, at which point the yield drops appreciably up to its maximum of 2.28 MeV (21). One potential issue associated with this high fluence of bremsstrahlung photons is the possibility of detector saturation.

Detector saturation due to the high singles rate from bremsstrahlung X-rays was initially thought to be a major barrier associated with ^90^ Y PET/CT. One of the first reports of successful clinical ^90^ Y PET/CT imaging (12) was performed with a custom fabricated 2.5-mm thick copper ring designed to reduce the singles rate due to bremsstrahlung from a patient dosage of only 1.3 GBq. Detector saturation at the dosages used in radioembolization has the potential to degrade image quality substantially and limit the quantitative accuracy of ^90^ Y PET – a major component of its utility.

## Image Quality

The first reported clinical ^90^ Y PET/CT scan (12) qualitatively demonstrated superior resolution to bremsstrahlung SPECT. Multiple authors have since published additional examples showing that ^90^ Y PET produces superior image quality when compared to alternative methods of imaging ^90^ Y such as bremsstrahlung SPECT or indirect imaging with ^99m^Tc MAA SPECT (17–19, 22–28).

### Resolution

**Figure 1:**
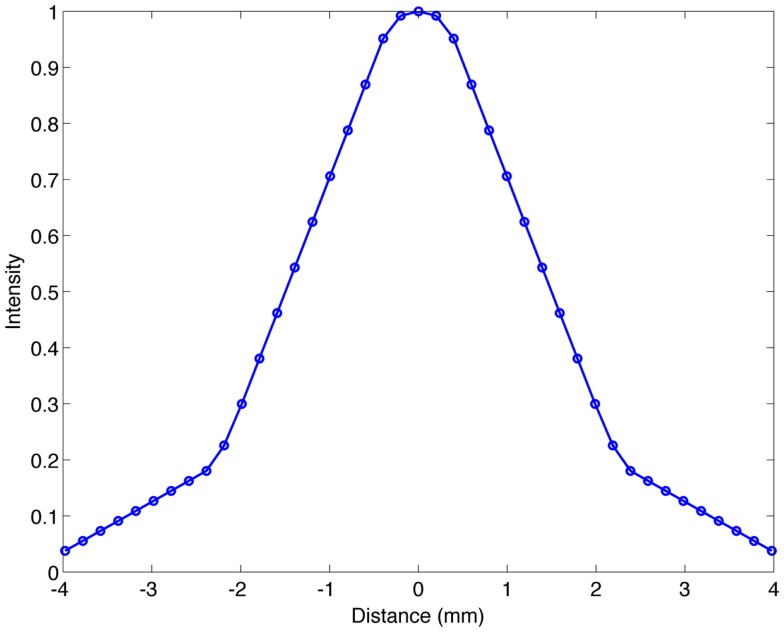
**Transaxial profile of a ^90^ Y line source in water with a FWHM of 3.1 mm**.

Resolution has been evaluated by a number of authors on many PET/CT systems. For ^90^ Y PET, the full-width at half-maximum (FWHM) has been reported to be anywhere between 5.0 (18) and 10.6 mm (15). Table 1 details the resolution reported in recent publications. It should be noted that the method used to evaluate resolution has varied from author to author, and this explains some of the inconsistencies seen. For example, Bagni et al. (17) and D’Arienzo et al. (18) both evaluated resolution on a GE Discovery ST and obtained a FWHM of 6.5 and 5.0 mm, respectively. We have evaluated the resolution of a Siemens BioGraph mCT Flow (Siemens Medical Solutions USA, Inc., Knoxville, TN, USA) using a 0.4-mm inner-diameter line source charged with ^90^ Y in a cylindrical water scatter phantom. The line source was positioned at isocenter. Reconstruction was performed using a 10-cm FOV (zoom of 8), a 512 × 512 PET reconstruction matrix, 1 iteration and 21 subsets with point spread function (PSF) resolution recovery (RR) and TOF. Under these conditions, the axial FWHM on this scanner was measured to be 3.1 mm. An axial profile of the line source from this acquisition is shown in Figure 1.

### Contrast

van Elmbt et al. (15) presented a detailed phantom analysis of ^90^ Y PET contrast using a custom built phantom. van Elmbt compared both the hot and cold contrast of varying size hot-spheres when filled with standard clinical activity concentrations in the spheres and background as a function of scanner type. A LYSO scanner (Philips Gemini TF, with and without TOF), a GSO scanner (Philips Gemini power 16), and a BGO scanner (Siemens ECAT Exact HR^+^) were all compared. Contrast was measured for effective scan times of 12, 24, 36, and 48 min on each scanner. The LYSO-based system with TOF produced the best hot contrast, regardless of scan time and/or sphere diameter. Results were somewhat different for cold contrast, where the intrinsic radioactivity of ^176^Lu adds to cold-sphere background and reduces nominal contrast when TOF was not used. The use of TOF compensated for some of this lost cold-sphere contrast, improving LYSO scanner performance. The BGO scanner was a poor performer, with a 40% loss of contrast for all sphere sizes and scan times, when both hot and cold spheres were analyzed (15). However, D’Arienzo et al. (18) was successful at using a BGO-based system to obtain contrast recovery exceeding 0.5 for 18 mm or larger spheres. D’Arienzo’s findings are similar to other publications where LSO and LYSO-based scanners were used (16, 19, 29).

Direct comparisons between ^90^ Y PET/CT and bremsstrahlung SPECT using identical scan time and similar reconstruction parameters have also been performed. Elschot et al. (19) reported contrast recovery to be significantly higher for phantom hot-spheres of all sizes using PET/CT on a Siemens mCT with TOF and RR when compared with a Siemens Symbia T16 with RR. In many cases, the contrast recovery produced using PET/CT exceeded bremsstrahlung SPECT by a factor of three or more. However, the ^90^ Y PET images had significantly more noise. In order to compare ^90^ Y PET and bremsstrahlung SPECT images with similar noise characteristics, reconstruction parameters of one iteration with a 15-mm Gaussian filter were applied to the PET data, producing similar noise to the bremsstrahlung SPECT. In this case, ^90^ Y PET still produced superior contrast (19). Elschot’s quantitative comparison of contrast recovery between PET and bremsstrahlung SPECT is in agreement with published patient results (17, 18, 23–28), which demonstrate this qualitatively.

van Elmbt et al. (15) analyzed PET/CT scans of a phantom filled with either ^90^ Y or ^18^F and found that the scatter fractions were nearly identical. Despite this finding, contrast recovery with ^18^F is still superior to ^90^ Y (15, 16, 19, 26). A clear, detailed analysis of this phenomenon is currently not present in the ^90^ Y PET literature, however there are several plausible explanations. The first can be attributed to the effect of high image noise on ordered-subset expectation maximization (OSEM) image reconstruction. Traditional OSEM reconstruction algorithms have an inherent non-negativity constraint, which may introduce a positive bias in low-count portions of the image. This same issue is present in dynamic PET where individual frames have high noise and special reconstruction methods are used to improve quantification and contrast recovery (30, 31). Another potential explanation for loss of contrast recovery is the possibility of pair-production events in the PET detector blocks from bremsstrahlung photons that exceed 1.022 MeV. van Elmbt et al. (15) suggested that these true-coincidence events may affect the ends of the profile tails of the rebinned sinograms. These pair-production events may have an impact on the scaling of the scattered sinogram to the emission sonogram and may impact randoms correction. Both of these phenomenon require future exploration as they relate to ^90^ Y PET.

### Noise

Yttrium-90 PET/CT images are inherently noisy due to the small positron emission branching fraction of ^90^ Y. Long clinical scan times of up to 45 min have been used to compensate for low true-coincidence rates in an effort to improve image quality (32). However, other authors have obtained acceptable image quality with scan times as short as 20 min (22). Further, while image quality may not be acceptable for clinical interpretation, dosimetry can be performed with scan times as low as 15 min (33).

**Figure 2:**
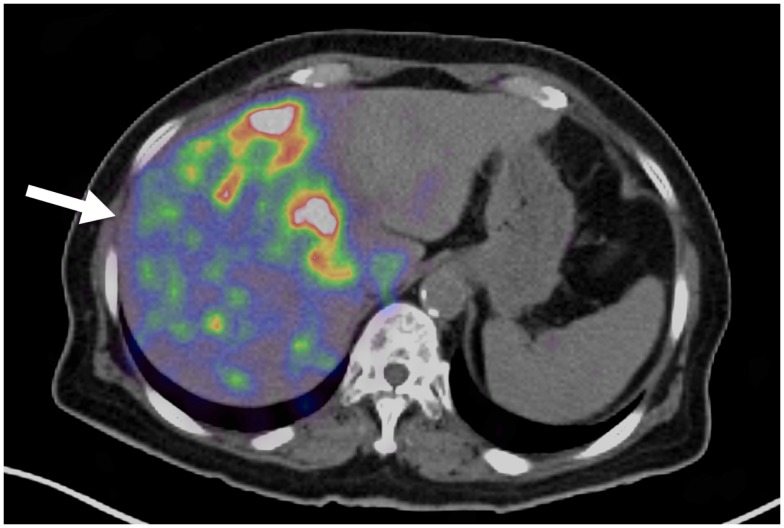
**Yttrium-90 PET/CT following radioembolization**. Note the non-uniformity present in the hepatic parenchyma (arrow).

The appearance of noise in clinical ^90^ Y imaging is often worse in the non-target hepatic parenchyma where the ^90^ Y activity concentration is lower, as illustrated in Figure 2. Although this low-level, clustered non-target activity is often attributed to quantum mottle, evidence suggests that this may represent a true physiologic phenomenon that can be visualized with PET/CT due to its high spatial resolution. Kennedy et al. (34) first observed clustering of microspheres in the pathologic analysis of four explanted livers following radioembolization. More recently, Walrand et al. (35) has provided simulation data supporting Kennedy’s findings using a virtual arterial tree model to simulate asymmetrical microsphere distribution.

## Quantification

### Linearity

Following Lhommel’s et al. (12) first report of successful clinical ^90^ Y PET/CT imaging with the use of a 2.5-mm thick copper attenuating ring to prevent detector saturation, it was thought that saturation might be the most significant factor preventing accurate quantification of ^90^ Y using PET/CT. However, since this initial report, numerous authors have presented results verifying count-rate linearity at the high activity levels common in radioembolization.

Detector saturation and non-linearity at clinical activities does not occur in PET systems that use detector blocks composed of LSO (16), LYSO, or GSO (15) due to their fast response time. Further, Lhommel followed up his initial report with a publication detailing count-rate linearity up to an activity of 2.2 GBq on the same scanner where a copper attenuator was initially used (13). On the far end of the spectrum, Willowson et al. (16) found excellent linearity up to an activity of 4.7 GBq on a Siemens BioGraph mCT. The limited geometric efficiency of PET due to solid angle is a major contributor to the lack of detector saturation. However, although bremsstrahlung SPECT has a higher geometric efficiency than PET, the poor efficiency of its collimator prevents saturation (15).

While a number of authors have found no evidence of detector saturation on LSO, LYSO, and GSO scanners, there is some controversy regarding saturation on BGO scanners. van Elmbt et al. (15) found evidence of non-linearity at activities >1.2 GBq on a Siemens Exact HR^+^ BGO PET system. Similarly, Walrand et al. (36) found detector saturation on a BGO system at activities above 1.5 GBq. van Elmbt concluded that BGO-based scanners are not suitable for ^90^ Y quantification, not due just to count-rate linearity issues but also the resultant 40% loss of hot contrast at clinical activity concentrations (15). However, it should be noted that the scanner used by van Elmbt et al. (15) was antiquated (Table 1) and newer BGO systems have shown to produce better results. Bagni et al. (17) found no evidence of saturation on a GE Discovery ST BGO PET system up to a total activity of 2.0 GBq. Similarly, D’Arienzo was able to obtain quantifiable images using a GE Discovery ST BGO scanner accurate to within 7.62% (18). Bagni et al. results suggest that some BGO detector systems may be acceptable for imaging many radioembolization procedures.

Table 1 details the maximum activity where count-rate linearity was found in several recent phantom studies, stratified by scanner type.

### Intrinsic radioactivity

The effect of intrinsic ^176^Lu radioactivity has been evaluated by assessing count-rate linearity and quantification accuracy at low total activity within the FOV. Goedicke et al. (33) found reasonable count-rate linearity on an LYSO-based system at total activities from 200 to 55 MBq (<16% error), suggesting that ^176^Lu provides a definite but not overwhelming contribution. Kao et al. (20) performed 15 phantom scans using available source vials with activities ranging from 3.5 MBq to 3.2 GBq placed in an acrylic scattering phantom. Across all imaged activities, the mean relative error in ^90^ Y PET quantification was 1.38% while vial activities exceeding 400 MBq had a mean error of only 0.29%. However, these analyses may not have been clinically realistic as they were performed using a decaying ^90^ Y source vial with a very small volume, preventing evaluation of activity concentration. While the total activity within the FOV was low, the activity concentration still may have been quite high.

Carlier focused on identifying the minimum detectable activity concentration (MDA) of ^90^ Y PET/CT (26). The MDA for a 10-mm hot-sphere in a 40:1 hot-sphere to background activity concentration was found to be ~1 MBq/mL with TOF and 3 MBq/mL without TOF. Spheres larger than 17 mm were detected at the lowest activity concentration imaged (0.49 MBq/mL). Similarly, Willowson et al. (16) found accurate quantification of large spheres and total activity within the FOV at activity concentrations as low as 0.380 MBq/mL. A study by Kao et al. (20) further corroborated these findings.

While accurate quantification at activity concentrations common in ^90^ Y radioembolization have been reported, one author designed a protocol specifically to quantify and subtract the signal resulting from intrinsic ^176^Lu activity (37). Using a two-step process, patients received a PET/CT scan both before and after the infusion of ^90^ Y using the same acquisition protocol. The pre-treatment scan served as a measurement of the background contribution from intrinsic ^176^Lu activity, allowing for later background subtraction. Data from an example patient presented showed an average pre-treatment activity concentration in the liver to be 29.6 Bq/mL, resulting from intrinsic ^176^Lu activity on a Siemens BioGraph 16 Truepoint PET/CT system. Interestingly, the average background found was significantly less than the typical tumor activity concentration in radioembolization, which is often >2 MBq/mL. This likely explains why other authors were able to obtain quantifiable results without the use of background subtraction.

### Reconstruction

Several publications have investigated optimal OSEM reconstruction parameters for quantifying ^90^ Y PET/CT. An in-depth analysis by Willowson et al. (16) examined the effect of reconstruction parameters on quantitative accuracy as a function of both hot-sphere size and activity concentration using the IEC NEMA Body Phantom. This phantom was initially imaged with a hot-sphere activity concentration of 3.9 MBq/mL and a background concentration of 0.47 MBq/mL. It was then serially imaged at multiple time points down to hot-sphere and background concentrations of 0.380 and 0.037 MBq/mL, respectively. Regardless of activity concentration or hot-sphere size, reconstruction parameters of 1 iteration and 21 subsets with TOF and RR provided the most accurate quantification on a Siemens BioGraph mCT. Carlier et al. (26) also performed a similar analysis and corroborated the conclusion that one iteration provides the most accurate quantification. It is likely that optimal reconstruction techniques are scanner-dependent as both Willowson and Carlier performed their analysis on Siemens BioGraph mCT systems, while D’Arienzo found two iterations to be optimal on a GE Discovery ST system (18). Goedicke et al. (33) used a Philips Gemini TF 16 system and found that the number of reconstruction subsets had a dominant effect on quantitative accuracy in both hot and cold regions, concluding that 2 iterations and 60 subsets was optimal. However, Goedicke noted that large numbers of subsets had the effect of increased noise clustering, perhaps negatively effecting uniformity or producing reconstruction-related artifacts.

There is overwhelming evidence in the literature to suggest that ^90^ Y PET is a quantifiable with a reasonable degree of accuracy. In all reported instances, neither detector saturation nor intrinsic ^176^Lu radioactivity substantially affected accurate quantification at activity concentrations common in radioembolization on modern scanners using LSO or LYSO detectors. Careful selection of reconstruction parameters can increase quantitative accuracy, however, the best choice will depend on the scanner and reconstruction software.

## ^90^Y PET/CT Dosimetry

The complexity of techniques for radioembolization treatment-planning and post-infusion dosimetry has grown significantly in the past 10 years. Beginning with the MIRD schema for ^90^ Y hepatic radioembolization (38), there has been an effort to estimate the dose to the tumor independently from the dose to the normal hepatic parenchyma. From compartmentalized models (38, 39), research in radioembolization has moved in the direction of 3-dimensional (3D) absorbed dose maps, which can be utilized for analysis of dose–volume histograms (DVH) and comparison of tumor-absorbed dose with known tumoricidal thresholds.

Image-based dosimetry for radioembolization has been performed in 3D using dose-point-kernel (DPK) convolution, with or without the use of ^90^ Y PET/CT. Both Kennedy et al. (40) and Dieudonne et al. (41) have used 3D SPECT data following infusion of ^99m^Tc MAA translated into absorbed dose maps using DPK convolution. A number of authors have used DPK convolution as a tool to compute absorbed dose in patient and phantom studies using post-infusion imaging with ^90^ Y PET/CT (13, 18, 19, 32, 37, 42). In several cases, DPKs were used from published literature such as reported by Strigari et al. (43) or Lanconelli et al. (44). However, because the DPK voxel size must match the PET acquisition matrix voxel size, DPKs were often calculated for specific scenarios using Monte Carlo radiation transport codes. Many Monte Carlo codes have been used in the ^90^ Y PET literature for this purpose including EGSnrc (40, 45), MCNP-X (19, 46), and FLUKA (37, 47).

Unlike other forms of targeted internal radionuclide therapy, radioembolization has the benefit of negligible biological removal following infusion. On that premise, absorbed dose following ^90^ Y radioembolization can be calculated by 3D convolution of the 3D activity concentration matrix with the β-DPK for ^90^ Y. Eq. 1 below describes this process:

(1) $$\begin{array}{llll} D\left(x,\;y,\;z\right) & =\frac{1}{\lambda }\left(A⊗\mathrm{DPK}\right)\left(x,\;y,\;z\right)\; & & \;\\ & =\frac{1}{\lambda }\sum_{z\prime }\sum_{y\prime }\sum_{x\prime }A\left(x\prime ,\;y\prime ,\;z\prime \right)*\mathrm{DPK}\; & & \;\\ & \;\times \left(x-x\prime ,\;y-y\prime ,\;z-z\prime \right) \end{array}$$

where λ is the decay constant of ^90^ Y, *A* represents the 3D activity concentration voxel space and DPK is the computed DPK per disintegration of ^90^ Y. In the reports cited above, the activity concentration matrix was taken from ^90^ Y PET/CT data. DPK convolution can be performed in a clinical setting using software such as the popular FDA approved MIDose code by DOSIsoft (Dosisoft, Cachan, France).

One alternative technique to DPK convolution that has been used in several ^90^ Y PET publications is a method whereby energy from ^90^ Y β-decay is deposited locally, within each voxel and no convolution is used. While this method may initially appear to be less accurate than DPK convolution, one must consider that the activity concentration matrix determined by PET/CT is not precisely *A* in Eq. 1, but rather *A* convolved with the PSF of the PET/CT system. On this premise, both Kao et al. (20) and Bourgeois et al. (48) have performed 3D dosimetry without using DPK convolution, under the assumption that the PSF of the PET/CT system approximates the ^90^ Y β-DPK. Kao et al. (20) presented justification for his technique in an included appendix, while Bourgeois et al. (48) cited a prior publication, which validated this method for SPECT (49).

## Clinical Utility of Post-Treatment ^90^Y PET/CT Imaging

^90^ Y PET/CT imaging following radioembolization serves in two primary clinical roles: (1) evaluating technical success and (2) predicting treatment efficacy.

### Evaluation of technical success

Successful radioembolization requires microsphere delivery within the target hepatic lobe and/or lesions selected for therapy. Although the primary goal of radioembolization is straightforward in principle, numerous procedural and physiologic factors contribute to, and potentially confound technical success. These include patient factors such as variable arterial hemodynamics and tumor vascularity, as well as technical factors such as catheter position, total particulate load delivered, and rate of microsphere delivery.

**Figure 3:**
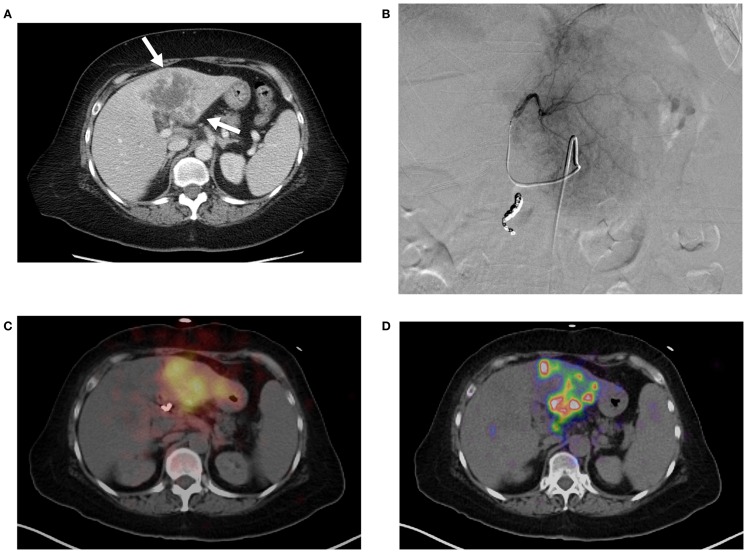
**(A)** Pre-treatment contrast enhanced CT imaging of metastatic cholangiocarcinoma in the left hepatic lobe. Areas of increased vascularity (arrow) are seen surrounding areas of necrosis. **(B)** Left hepatic angiogram during infusion of ^90^ Y into left lobe. **(C)** Bremsstrahlung SPECT following radioembolization demonstrates diffuse activity in the region of the left lobe hepatic neoplasm. **(D)** ^90^ Y PET/CT demonstrates more detailed information with multifocal areas of maximum activity corresponding to the peripheral viable tumor on pre-treatment imaging.

Technical success with ^90^ Y trans-arterial hepatic embolization has traditionally been assumed by successful infusion of ^90^ Y into the target artery and bremsstrahlung SPECT demonstrating activity in the region of the targeted hepatic lobe. However, accurate confirmation of pre-treatment radiation-planning intentions was not possible using conventional bremsstrahlung SPECT, as illustrated in Figure 3. Figure 3A shows the pre-treatment hepatic protocol CT obtained on a patient with metastatic cholangiocarcinoma in the left lobe. The neoplasm is characterized by central necrosis surrounded by peripheral contrast-enhancing viable tumor. The left hepatic arteriogram in Figure 3B confirms infusion of SIRTeX SIR-Sphere^®^ radioembolization into the target artery. Finally, the post-treatment bremsstrahlung SPECT in Figure 3C demonstrates diffuse activity in the region of the left lobe hepatic neoplasm. However, due to the limited resolution of bremsstrahlung SPECT, a detailed comparison with pre-treatment imaging was not possible. The post-treatment ^90^ Y PET/CT in Figure 3D demonstrates more detailed information with multifocal areas of maximum activity corresponding to the peripheral viable tumor seen on the pre-treatment hepatic CT.

**Figure 4:**
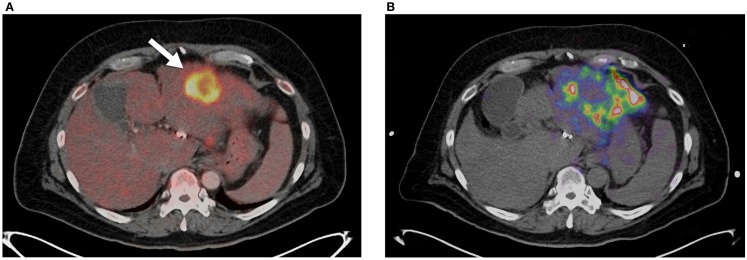
**(A)** ^18^FDG PET/CT showing large focal lesion from metastatic cholangiocarcinoma prior to radioembolization (arrow). This was the only conspicuous lesion in this patient’s left lobe. **(B)** ^90^ Y PET/CT following radioembolization. Poor tumor uptake can be seen (T/N <1) with the majority of the dosage distributing to non-target areas of the left lobe.

Lack of technical success can also be identified using ^90^ Y PET/CT with high specificity. Historically, lack of technical success would not be apparent until delayed conventional imaging and clinical follow-up determine poor success in tumor management. Identification of technical failure on ^90^ Y PET/CT imaging gives the opportunity for treating physicians to consider additional therapies before discovering clinical failure weeks to months later. Alternative or supplemental therapies such as trans-arterial drug eluting bead embolization or percutaneous thermal ablation may be considered in a timely manner. Figure 4 illustrates an example of technical failure as compared to pre-treatment radioembolization therapy-planning intent. Figure 4A is a pre-treatment ^18^FDG PET/CT with conspicuous tumor activity in the left hepatic lobe. Technical success was initially inferred from successful infusion of the SIRTeX SIR-Sphere^®^ radioembolization dosage into the left hepatic artery, and presence of left lobe activity on the post-treatment ^90^ Y bremsstrahlung SPECT scan. However, the ^90^ Y PET/CT following radioembolization demonstrated lack of significant uptake in the region of most active tumor (Figure 4B). A detailed description of ^90^ Y PET/CT diagnostic reporting, including many more examples has been presented by Kao et al. (27). Many other reports have recently presented results and example cases comparing bremsstrahlung SPECT post-treatment images with ^90^ Y PET/CT images, illustrating qualitatively superior resolution and contrast with ^90^ Y PET/CT (12, 22–26, 28).

### Quantitative prediction of treatment efficacy

^90^ Y PET/CT equips treating physicians with precise information regarding the heterogeneity of microsphere distribution within a lesion and the absorbed radiation dose to each part of the lesion. These factors could be helpful in predicting treatment response or the need for early triage to alternative or adjuvant therapy. Several authors have used ^90^ Y PET-based dosimetry to predict treatment response in patients receiving radioembolization (18, 20, 32, 42, 48). However, while there are standardized and accepted methods for generating 3D absorbed dose maps from ^90^ Y PET/CT data, there is no standardization of the dose metric used to predict treatment response in radioembolization patients.

Chang et al. (32), Bourgeois et al. (48), and D’Arienzo et al. (42), all used the average absorbed dose to the lesion (*D*_avg_). In an article by Bourgeois et al. (48), *D*_avg_ was selected to coincide with one of the largest published patient studies detailing radiobiologic toxicity of SIRTeX SIR-Sphere^®^ radioembolization in hepatocellular carcinoma (HCC) (50). Alternatively, Kao et al. (20) used *D*_70_ and *V*_100_ values for tumor analysis, where *D*_70_ and *V*_100_ are the minimum absorbed dose delivered to 70% of the tumor volume and *V*_100_ is the percentage of tumor volume exceeding 100 Gy. Kao et al. (20) outlines several applicable external beam radiotherapy standards which could be applied to ^90^ Y radioembolization.

The missing component for the accurate prediction of treatment efficacy using ^90^ Y PET/CT following radioembolization is a detailed compendium of radiobiologic toxicities. The ideal minimum requirements for such a database would include dose–response data that is stratified by the type of radioembolization used (i.e., SIRTeX SIR-Spheres^®^ or BTG ThereSpheres^®^) and the type of tumor being treated. However, no such database currently exists. Strigari has published (50) an excellent database compiled from 73 patients treated for HCC with SIRTeX SIR-Spheres^®^ and her data remains one of the most complete offerings in the current literature. However, because ^90^ Y PET/CT can provide quantitative post-treatment imaging, it is likely that more dose–response data will be available in the near future. As these data become available, clinicians who choose to use it along with post-treatment Y^90^ PET to predict treatment efficacy or modify treatments (as described in the next section) must be particularly cautious that the referenced dose–response data is appropriate for the clinical case. For instance, application of Strigari’s dose–response data (50) to the clinical decision making process for a patient treated with BTG ThereSpheres^®^ would be contraindicated. While ^90^ Y PET can quantify absorbed dose from SIRTeX SIR-Spheres^®^ or BTG ThereSpheres^®^ with equal accuracy, due to the activity per microsphere as well as the number of microspheres used, the dose–response relationship between the two products is significantly different.

## ^90^Y PET/CT Imaging as a Tool for Improving Treatment

In addition to confirmation of technical success and prediction of treatment efficacy, ^90^ Y PET/CT imaging has the potential to prospectively improve therapy. Recent reports explore two mechanisms by which ^90^ Y PET/CT can be used to directly tailor treatment-planning for radioembolization.

The current advanced method of ^90^ Y treatment-planning involves use of ^99m^Tc MAA as a microsphere surrogate. The partition model (39) is used clinically as a radioembolization treatment-planning tool at many sites based on the measurement of T/N on ^99m^Tc MAA SPECT imaging. However, factors including differences in catheter position, embolic load, particle size, injection technique, free ^99m^Tc, and physiologic changes occurring between MAA and radioembolization procedures may affect the accuracy of MAA as a microsphere surrogate. Knesaurek et al. (51) suggested that these factors explained varying agreement found between the hepatic distribution of MAA and radioembolization in a 20-patient study. Wondergem et al. (52) also reported poor correlation between MAA and ^90^ Y distribution. However, Kao et al. (20) found good agreement between dosimetry based on ^99m^Tc MAA and ^90^ Y PET.

While debate on the effectiveness of MAA as a radioembolization surrogate still exists, several recent reports have incorporated ^90^ Y PET/CT into treatment-planning algorithms to optimize therapy without relying on ^99m^Tc MAA SPECT imaging. Chang et al. (32), presented a report where a patient received two separate SIRTeX SIR-Sphere^®^ radioembolization treatments for cholangiocarcinoma metastases in the right and left hepatic lobes. The SIRTeX body surface area (BSA) treatment-planning model was used for the treatment of the right hepatic lobe lesion. Following the first treatment, ^90^ Y PET indicated that the absorbed dose to the right lobe tumor was <70 Gy with a T/N measuring just 2.5:1. Subsequent radioembolization of a lesion in the left hepatic lobe was performed with treatment-planning based on the partition model (39) and the T/N measured from the ^90^ Y PET/CT of the right lobe. Another ^90^ Y PET/CT performed after the left lobe treatment indicated a tumor-absorbed dose of 110 Gy. Follow-up imaging demonstrated no response in the right lobe lesion and complete response in the left lobe (32).

While debate exists on the effectiveness of MAA as a radioembolization surrogate, one can conclude that Chang’s method (32) did eliminate some uncertainties in that it did not rely on MAA. However, Chang applied measurements from focal disease in one lobe to treat focal disease in another lobe, which has unproven validity (32). A subsequent report by Bourgeois et al. (48) described results from a patient with HCC who underwent a novel dual-infusion, single-day radioembolization protocol using SIRTeX SIR-Spheres^®^. An initial ^90^ Y infusion was performed using a conventional treatment-planning model, followed by ^90^ Y PET/CT. A 3D absorbed dose map was generated from ^90^ Y PET, which allowed for quantification of a tumor-absorbed dose of 53.2 Gy. A simple arithmetic proportion was then applied to determine the necessary additional ^90^ Y dosage required to achieve a total tumor-absorbed dose of 120 Gy, corresponding to published recommendations for HCC (50). The additional dosage was delivered in the same day, with comparable catheter placement and resulted in a robust treatment response (48). The benefit to Bourgeois’ protocol was that it provided patient-specific radioembolization treatment-planning, however, the complexity of the protocol may limit clinical use.

There may be utility in adapting Bourgeois’ protocol (48) as part of a multi-cycle fractionated radioembolization treatment method, previously discussed by Cremonesi et al. (53). This would allow patient-specific treatment-planning to be combined with increased sparing of normal liver parenchyma, increased dose to the tumor, and may be a potential future application of ^90^ Y PET/CT.

## Improving ^90^Y PET/CT Imaging

Multiple authors have found excellent quantification, resolution, and contrast recovery in ^90^ Y PET/CT phantom studies. However, there is still potential for improving both quantitative accuracy and image quality in ^90^ Y PET/CT. As previously discussed, contrast recovery for ^90^ Y PET is less than ^18^F PET. Because the scatter fractions have been found to be nearly identical (15) for ^18^F and ^90^ Y, it is likely that the differences in contrast recovery may be attributed to the effects of image noise on conventional reconstruction algorithms. Standard clinical OSEM reconstruction algorithms contain an inherent non-negativity constraint, which is appropriate for conventional radiotracers where image noise is low. However, this non-negativity constraint can contribute to a positive bias in ^90^ Y PET images, particularly in the low-count background resulting in a decrease in contrast recovery. A similar issue has been found in dynamic PET, where short duration frames contribute to high image noise resulting in a positive bias when conventional OSEM is used (30). NEG-ML (54) and AB-EM (55) are two reconstruction algorithms without non-negativity constraints that have been used to address this issue in dynamic PET. Verhaeghe and Reader (30) have compared both algorithms, and while either improves quantification accuracy in dynamic PET compared to conventional OSEM, AB-EM produced the best results. Application of these techniques to ^90^ Y PET may, therefore, provide a benefit. However, widespread use will likely require direct support from PET/CT manufacturers.

Another area where ^90^ Y quantification can be improved is with application of gated acquisition protocols. Several studies have shown a quantitative improvement with gated ^18^FDG PET. Lupi et al. (56) performed non-gated and gated PET/CT studies in 22 patients with lung carcinoma. Gating resulted in a maximum average increase of 77% in the SUV of measured lesions. Werner et al. (14) evaluated the effect of respiratory gating during ^18^FDG PET/CT on estimation of lesion size and SUV in 18 patients with pulmonary nodules and found a 22% maximum average increase in SUV, as well as a decrease in nodule volume by 44.5%. Gating has also shown to be useful for ^18^FDG PET/CT imaging of liver metastases, mirroring results from chest studies with statistically significant increases in SUV with the use of a respiratory-gated protocol (57). However, traditional phase-based gating used in PET is based on principles applied to cardiac gating, where a temporal description of motion is useful for diagnosis. The PET dataset is divided into multiple temporal bins with the phase of the breathing cycle. This process results in a significant decrease in the number of counts contributing to each image or bin, increasing image noise. Due to the low true-coincidence count-rate associated with ^90^ Y PET, phased-based gating is not a viable option since the resulting images would be too noisy to be quantitatively or diagnostically useful.

Amplitude-based gating is an alternative to phase-based gating first described in 4D-CT by Rietzel et al. (58) and further refined to account for motion by Wink et al. (59). This method uses amplitude information rather than temporal information to sort the reconstructed data. Amplitude-based methods were applied to PET/CT imaging by Wang et al. (60), who described the performance of this type of algorithm in controlled phantom studies. This gating method is useful in PET/CT imaging as it retains more counts than phase-based gating with the trade off of a loss of a temporal description of motion.

**Figure 5:**
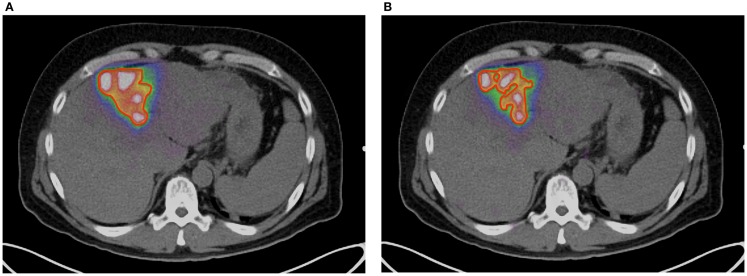
**(A)** ^90^ Y PET of a patient with HCC. Scan performed on Siemens BioGraph mCT Flow with TOF and RR. A continuous bed speed of 0.2 mm/s, 1 iteration, and 21 subsets were used for reconstruction. The contour (red) was performed by an automatic segmentation tool with the lower threshold set to an absorbed dose of 100 Gy. **(B)** ^90^ Y PET of a patient with HCC. Scan performed on Siemens BioGraph mCT Flow with TOF, RR, and optimal respiratory gating (HD-Chest). A continuous bed speed of 0.2 mm/s, 1 iteration, and 21 subsets were used for reconstruction. The contour (red) was performed by an automatic segmentation tool with the lower threshold set to an absorbed dose of 100 Gy.

A method of amplitude-based gating, known as optimal respiratory gating, was developed in 2010 by Hamill et al. and patented in 2011 (61). This method uses an upper and lower threshold pair correlated to a measured respiratory signal to create an optimally gated static PET image that corresponds to the phase of the respiratory cycle where motion is at a minimum. In recent work, the authors of this manuscript have performed ^90^ Y PET/CT scans with continuous bed motion acquisition techniques (FlowMotion) in conjunction with optimal respiratory gating (HD-Chest) using a Siemens BioGraph mCT Flow (Siemens Medical Solutions USA, Inc., Knoxville, TN, USA). Initial results show decreases in visualized tumor volume and increases in ^90^ Y activity concentration with the use of optimal respiratory gating techniques. Figures 5A,B qualitatively illustrate the difference between an acquisition performed on a Siemens BioGraph mCT Flow with TOF and RR, with and without HD-Chest. Using automatic contouring with the same threshold, visual tumor volume decreased by 33% when optimal respiratory gating is used, along with increases in average and maximum activity concentration. Scan times on the order of 25 min produce acceptable image quality, even with the gated protocol (Figure 5B).

## Conclusion

^90^ Y PET/CT is a tool that has the potential to contribute significantly toward improving radioembolization in the future. Accurate quantification of activity concentration has been demonstrated on a variety of PET/CT systems, with or without TOF. Further, methods are available to transform ^90^ Y PET image sets into 3D absorbed dose maps in the setting of a traditional clinical workflow. In fact, many institutions with a modern PET/CT system are currently fully equipped to perform post-treatment ^90^ Y PET/CT scans and generate absorbed dose maps. Not only will post-treatment ^90^ Y PET/CT allow treating physicians to more appropriately manage the care of their patients, but also increasing utilization of ^90^ Y PET has the potential to provide a wealth of dose–response information. This information may lead to the development of improved radioembolization treatment-planning models in the future.

## Conflict of Interest Statement

The authors declare that the research was conducted in the absence of any commercial or financial relationships that could be construed as a potential conflict of interest.
